# N,N-dimethyltryptamine effects on connectome harmonics, subjective experience and comparative psychedelic experiences

**DOI:** 10.1038/s41386-025-02190-4

**Published:** 2025-09-12

**Authors:** Jakub Vohryzek, Andrea I. Luppi, Selen Atasoy, Gustavo Deco, Robin L. Carhart-Harris, Christopher Timmermann, Morten L. Kringelbach

**Affiliations:** 1 Centre for Eudaimonia and Human Flourishing, Linacre College, University of Oxford, Oxford, United Kingdom; 2 Center for Brain and Cognition, Computational Neuroscience Group, Department of Information and Communication Technologies, Universitat Pompeu Fabra, Barcelona, Spain; 3 Department of Psychiatry, University of Oxford, Oxford, United Kingdom; 4 St John’s College, University of Cambridge, Cambridge, United Kingdom; 5 Division of Information Engineering, University of Cambridge, Cambridge, United Kingdom; 6 Center for Music in the Brain, Aarhus University, Aarhus, Denmark; 7 Institucio Catalana de la Recerca i Estudis Avancats (ICREA), Passeig Lluis Companys 23, Barcelona, Spain; 8 Centre for Psychedelic Research, Department of Brain Sciences, Imperial College London, London, United Kingdom; 9 Departments of Neurology and Psychiatry, University of California San Francisco, San Francisco, CA USA; 10 Department of Experimental Psychology, University College London, London, UK

**Keywords:** Neuroscience, Diagnostic markers, Biological techniques

## Abstract

Exploring the intricate relationship between brain’s structure and function, and how this affects subjective experience is a fundamental pursuit in neuroscience. Psychedelic substances offer a unique insight into the influences of specific neurotransmitter systems on perception, cognition and consciousness. Specifically, their impact on brain function propagates across the structural connectome — a network of white matter pathways linking different regions. To comprehensively grasp the effects of psychedelic compounds on brain function, we used a theoretically rigorous framework known as connectome harmonic decomposition. This framework provides a robust method to characterize how brain function intricately depends on the organized network structure of the human connectome. We show that the connectome harmonic repertoire under N,N-dimethyltryptamine (DMT) is reshaped in line with other reported psychedelic compounds - psilocybin, lysergic acid diethylamide (LSD) and ketamine. Furthermore, we show that the repertoire entropy of connectome harmonics increases under DMT, as with those other psychedelics. Importantly, we demonstrate for the first time that measures of energy spectrum difference and repertoire entropy of connectome harmonics index the intensity of subjective experience of the participants in a time-resolved manner reflecting close coupling between connectome harmonics and subjective experience.

## Introduction

Understanding how subjective experience arises from the dynamic interplay of brain structure and function is a central question in neuroscience. In combination with non-invasive neuroimaging, psychedelic substances offer a powerful window to interrogate how specific neurotransmitter systems shape brain function to influence perception, cognition, and consciousness [1].

Crucially, the changes in brain function exerted by neurotransmitter engagement propagate throughout the brain according to the network of white matter pathways between regions: the human structural connectome [2]. Therefore, understanding the effects of psychedelic compounds on brain function involves bridging structure and function across multiple levels [3, 4].

A theoretically rigorous way to characterise how brain function depends on the underlying network organisation of the human connectome is provided by the framework of connectome harmonic decomposition (CHD) [5, 6]. Mathematically, CHD represents functional signals in terms of their dependence on the intrinsic modes of the underlying structural connectome – the connectome harmonics (CHs). In other words, CHs are a change of basis functions, analogous to the Fourier transform that transforms a signal from the time domain into the domain of temporal frequencies. Likewise, CHD transforms brain signals from the spatial domain, into the domain of connectome frequencies. CHD explicitly expresses brain activity in terms of multi-frequency contributions from the underlying structural network: each connectome harmonic is a distributed activation pattern characterized by a specific spatial scale (frequency). Low-frequency (coarse-grained) connectome harmonics indicate that the functional signal reflects global connectivity patterns in the underlying structural connectome. In turn, high-frequency (fine-grained) connectome harmonics indicate a divergence between the spatial organisation of the functional signal coupled to the (coarse-grained) underlying network structure: nodes may exhibit different functional signals even if they are closely connected to the same structural network [7]. The implementation of the decomposition of cortical activity into connectome-specific harmonics reflects the contribution of structural organization to brain activity across different spatial scales of resolution, and hence extends on and goes beyond previous investigations that considered the structure-function relationship of brain organisation at a single scale [8–11].

Recent work has consistently demonstrated two prominent effects of psychedelics on the connectome harmonic landscape of the human brain. First, the serotonergic psychedelics, LSD and psilocybin, as well as the atypical psychedelic, ketamine, consistently induce a reduction in the contribution of low-frequency (large-scale) harmonics, and a corresponding increase in the contribution of high-frequency (fine-grained) harmonics [5, 12, 13]. This evidence is also in line with additional reports of LSD-induced structure-function decoupling [14] where others have interpreted a shift away from low-frequency harmonics in favour of high-frequency ones as decoupling of brain activity from the underlying structural connectivity [7]; or at least from the major white-matter tractography. Second, psychedelics induce a broadening of the repertoire of connectome harmonics that contribute to spontaneous brain activity [13] alongside evidence of increases in the spatio-temporal metastability of brain function in the psychedelic state [15, 16].

Here, we hypothesise that as a potent serotonergic psychedelic, DMT will reshape the connectome harmonics in line with the effects previously reported for LSD and psilocybin, as well as the atypical psychedelic, ketamine. Namely, we predict a decreased contribution from low-frequency harmonics under the effects of DMT, and instead an increase in the contribution of high-frequency harmonics. We also hypothesise that like other psychedelics, DMT will increase the diversity (entropy) of the repertoire of connectome harmonics.

A crucial feature of the effects of intravenous (IV) DMT, that makes it especially valuable for scientific investigation is that, whereas oral LSD- and psilocybin-induced effects have a slow onset and can last for several hours, the effects of IV DMT are relatively more contracted and temporally predictable. IV DMT has a fast onset and reliably short duration of ~8 min for the dosage and injection parameters used here [17, 18]. This feature of DMT makes it possible to obtain dynamic ratings of the intensity of subjective experience over time and then relate these data to the corresponding time-resolved changes in connectome harmonics - since CHD analysis is also applicable on a dynamic timepoint-by-timepoint basis. Recent results have shown that neural changes in connectome harmonic signature reflect changes in subjective experience [5, 13]. However, those results were time-averaged across the entire scan duration. Therefore, here we capitalise on the unique temporal resolution offered by DMT to test a stronger hypothesis: that the neural changes in connectome harmonic composition - as described by energy spectrum difference and repertoire entropy - will be related to behavioural changes in intensity ratings, not just on average, but rather in a dynamic timepoint-by-timepoint manner, reflecting close coupling between connectome harmonics and subjective experience.

## Methods

### DMT dataset

The complete description of the participants, the experimental design and the acquisition parameters can be found in [17, 18]. All participants gave written informed consent to take part in the study. Ethical approval was granted by the National Research Ethics Committee London—Brent and the Health Research Authority. The study adhered to the revised Declaration of Helsinki (2000), the International Committee on Harmonization Good Clinical Practice guidelines, and the National Health Service Research Governance Framework. Sponsored by Imperial College London, the research was conducted under a Home Office license for studies involving Schedule 1 drugs.

In the following, we provide a succinct account of consistent information. For Psilocybin [19] and LSD [20] datasets, we provide details in the Supplementary Information together with methodological details pertaining to the Connectome Harmonics framework.

### Participants

A group of 25 participants was recruited in a single-blind, counter-balanced and placebo-controlled design. Participants underwent physical and mental health screening, which included a routine physical examination, electrocardiogram (ECG), blood pressure and pulse measurement, routine blood tests, and a psychiatric interview conducted by a medical professional. The main exclusion criteria were: being under 18 years of age, no prior experience with a psychedelic or hallucinogenic drug, a personal history of diagnosed psychiatric illness, an immediate family history of psychotic disorders, excessive alcohol use (more than 40 units per week), and a phobia of blood or needles. In addition, participants were required to complete a urine test for drugs of abuse and, where applicable, for pregnancy. Out of the 25 participants 20 completed the whole study (7 female, mean age = 33.5 years, SD = 7.9). A further 3 subjects were excluded due to excessive motion during the 8 min DMT recording (more than 15% of volumes scrubbed with framewise displacement (FD) of 0.4 mm). The final count of 17 participants is consistent with the previously published work on the DMT dataset by Vohryzek et al. [21]. For the time-resolved analysis of Fig. 5 further 3 subjects were removed due to excessive motion (>20% of scrubbed volumes with a FD threshold of 0.476).

### Experimental paradigm

**Fig. 1:**
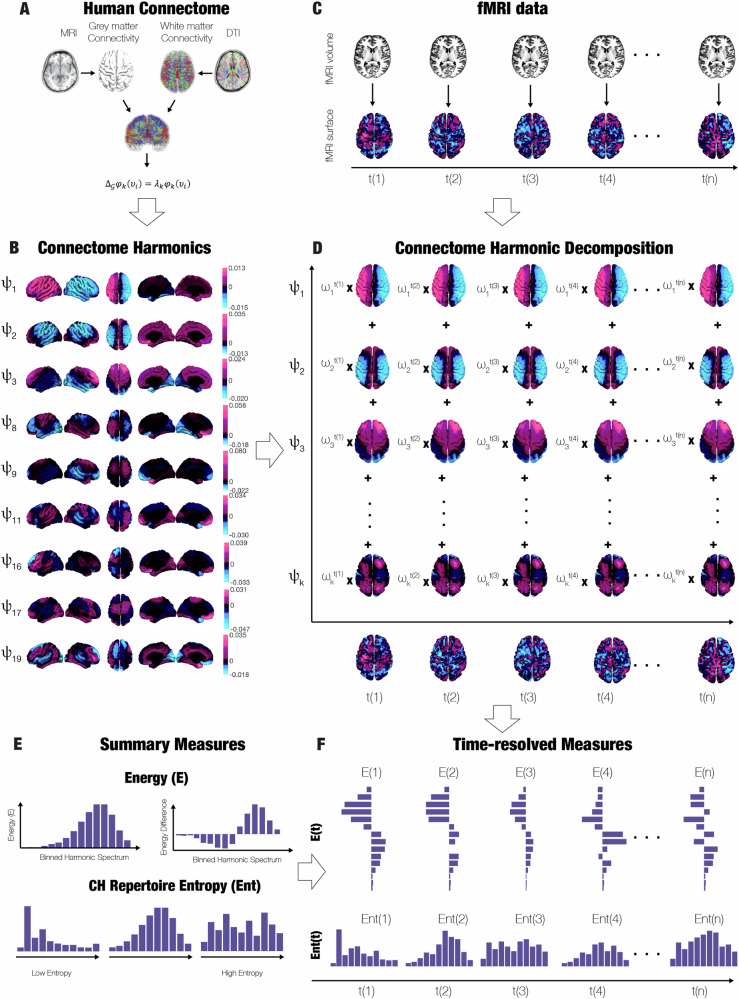
Study overview. **A** Human Connectome constructed from structural and diffusion MRI. **B** Connectome Harmonics computed from the eigendecomposition of Laplacian operator applied to the human connectome. **C** Functional MRI projected from MNI volumetric space to Freesurfer surface space. **D** Connectome Harmonic Decomposition summarising fMRI timeseries as a linear summation of individual harmonics and their weights. **E** Summary measures for interpreting the Connectome Harmonic Decomposition. Namely, the energy spectrum, energy spectrum difference and CH repertoire entropy. **F** Time-resolved measures applied to the entire course of the fMRI recording.

In total, all subjects were scanned on two days, two weeks apart, each consisting of two scanning sessions. The initial scan lasted 28 min with the 8th minute marking the intravenous administration of either DMT or placebo (saline) (50/50 DMT/placebo), single bolus lasting 60 s. Subjects were asked to lay in the scanner with their eyes closed (wearing an eye-mask). After the recording, assessment of subjective effects was carried out. The second session was identical to the first except for the assessment of subjective intensity scores at every minute of the recording. The experimental design also included simultaneous EEG recording during the sessions (see Fig. 1).

### fMRI acquisition parameters

The experiment was performed on a 3T scanner (Siemens Magnetom Verio syngo MR 12) with compatibility for EEG recording. A T2^∗^-weighted echo planar sequence was used. In brief, the parameters were as follows: TR/TE = 2000ms/30 ms, acquisition time = 28.06 min, flip angle = 80°, voxel size = 3 × 3 × 3 mm^3^ and 35 slices with 0 mm interslice distance. T1-weighted structural scans of the brain were also acquired.

### fMRI pre-processing

For fMRI pre-processing, a pipeline previously developed for an LSD experiment was used, which can be accessed in the supplementary information of [20]. Briefly, the following steps were applied 1) despiking, 2) slice-timing correction, 3) motion correction, 4) brain extraction, 5) rigid body registration to structural scans, 6) non-linear registration to 2 mm MNI brain, 7) motion-correction scrubbing, 8) spatial-smoothing (FWHM) of 6 mm, 9) band-pass filtering into the frequency range 0.01–0.08 Hz, 10) linear and quadratic detrending, 11) regression of 9 nuisance regressors (3 translations, 3 rotations and 3 anatomical signals).

### Structural connectome construction

For the construction of group connectome harmonics, an independent cohort of 10 participants (6 female, 22–35 years) was used from the Human Connectome Project (HCP), WU-Minn Consortium (Principal Investigators: David Van Essen and Kamil Ugurbil: 1U54MH091657). This project was made possible by funding from the sixteen NIH Institutes and Centres supporting the NIH Blueprint for Neuroscience Research; and by the McDonell Centre for Systems Neuroscience at Washington University. Both structural and Diffusion Tractography Imaging (DTI) data was used for the construction of connectomes with pre-processing according to the minimal pre-processing guidelines of the HCP protocol [22].

For the estimation of the connectome harmonics, we used the identical workflow as in Atasoy et al. [5]. In general, this consisted of combining local, surface based, and long-range white- matter connectivity in a sparse vertex-based representation. In brief, cortical surface reconstruction from high-resolution T1-weighted MRI of individual participants was carried out with Freesurfer software. Then, each participant’s cortical surface was registered to the 1000-subject group template yielding a common-space mesh of 10,242 vertices in each hemisphere. For the white-matter cortico-cortical fibres, deterministic tractography was applied to the DTI data of individual subjects (resolution 1.25 mm) with Matlab implementation of Vista Lab, Stanford University. For the tractography itself, eight seeds were initialised in each vertex (total of 20,484) with the termination criteria being either fractional anisotropy (FA) below 0.3, minimum track length of 20 mm and a maximum angle of 30° between two adjacent tracking steps.

It is to be noted, that the main analysis is carried out using the aforementioned structural connectome reconstruction to allow for consistency with previously reported results using CHD on psilocybin [12] and LSD [5]. In the Supplementary Information to ensure robustness and reproducibility of the results, we further report an alternative structural connectome reconstructed from multi-shell diffusion-weighted imaging data from 985 subjects of the HCP 1200 data release. Lastly, the derivation of the group-averaged structural connectomes is ultimately based on the assumption that the fundamental bases, here referred to as connectome harmonics, are consistent building blocks across participants. Indeed, recent work has demonstrated that, group-averaged information at the white-matter connectivity and cortical folding level can reconstruct well both spontaneous and task-evoked fMRI activity [23, 24].

## Results

Using connectome harmonics as the spatial basis of brain activity, it is possible to describe the temporal evolution of connectome harmonics in terms of their contribution. Here, we use CHD to describe the spatio-temporal changes of the DMT-induced state in terms of its connectome harmonic spectrum and repertoire diversity (entropy).

### The DMT-induced state suppresses low-level harmonics and increases high-level harmonics

We first estimated the connectome harmonic energy spectrum of each condition (DMT pre/post and PCB pre/post) across all timepoints and subjects. Following the established procedure for connectome harmonic analysis [5, 12], we then binned the connectome harmonic spectrum into 15 logarithmically spaced bins and obtained the harmonic profiles.

**Fig. 2:**
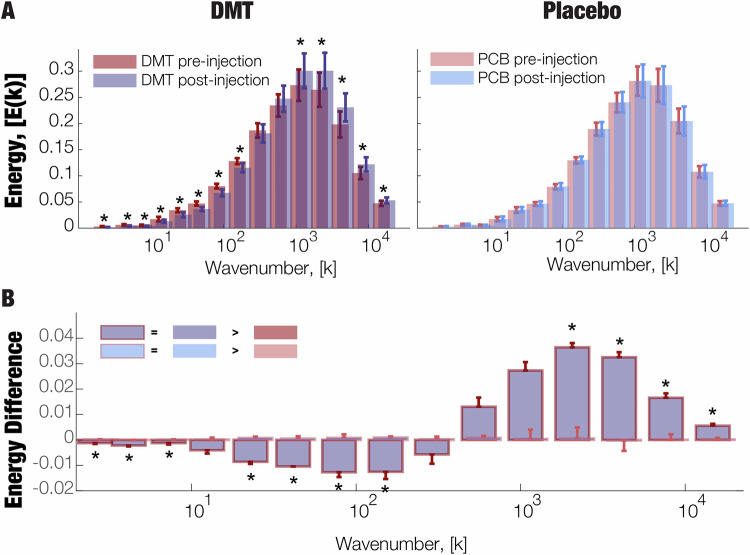
DMT-induced energy changes in the connectome harmonic spectrum. **A** A decrease of energy for low-frequency harmonics *k ∈* [1,…,10^2^] (*p-value < 0.01, Bonferroni corrected paired *t*-test) and an increase of energy for high-frequency harmonics *k ∈* [10^3^,…,10^4^] (*p-value < 0.01, Bonferroni corrected paired t-test) were observed. No significant changes were observed in the placebo conditions. **B** Furthermore, energy differences between the DMT and placebo conditions were observed with decreases in low-frequency harmonics *k ∈* [1,…,10^2^] (*p-value < 0.01, Bonferroni corrected paired *t*-test) and increases in high- frequency harmonics *k ∈* [10^3^,…,10^4^] (*p-value < 0.01, Bonferroni corrected paired *t*-test). No significant differences were observed at the inflexion point [10^2^,…,10^3^] (see Supplementary Table S1 for a full report of the *p*-values).

For the DMT-induced state, a range of low frequency harmonic bins (*k ∈* [1,…,10^2^]) were found significantly suppressed as opposed to the pre-DMT condition (*p*-value < 0.01, Bonferroni corrected paired *t*-test). No significant differences were observed in the placebo condition. A mirror opposite change was observed in the high frequency harmonic bins, whereby a range of *k ∈* [10^3^,…,10^4^] was found significantly increased (*p*-value < 0.01, Bonferroni corrected paired *t*-test). Again, no significant differences were observed for the placebo condition (Fig. 2A). This profile change across quantized harmonic bins can be further explored by looking at the energy differences across the DMT conditions of each subject while comparing it to the placebo condition difference. Remarkably, a similar suppression of the lower-harmonics *k ∈* [1,…,10^2^] (*p*-value < 0.05,

Bonferroni corrected paired *t*-test) and an increase of the higher harmonics *k ∈* [10^3^,…,10^4^] (*p*-value < 0.01, Bonferroni corrected paired *t*-test) are observed with non-significant results for bins at the inflexion point [10^2^,…,10^3^] (Fig. 2B). For the full list of the p-values for the related comparisons please consult Supplementary Table S1. Furthermore, these energy distribution changes of CH under DMT are robust to the choice of connectome as the results are consistent with the analysis performed with the 985 HCP participant connectome (Supplementary Fig. 1). Lastly, these energy distribution changes are also consistent when comparing the DMT and placebo post-injection conditions for both the original and 985 HCP participant connectomes (Supplementary Fig. 2).

### Contextualising DMT-induced changes in connectome harmonic spectrum against other states of consciousness

**Fig. 3:**
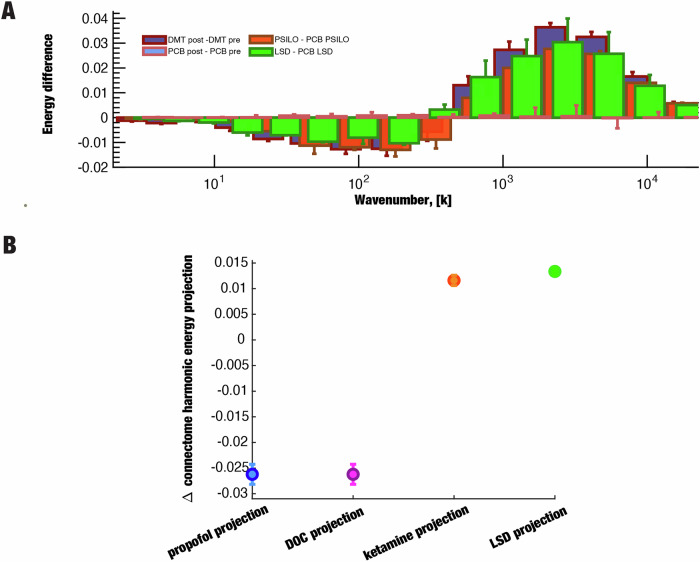
Contextualising the Connectome Harmonic signature of DMT with other altered brain states. **A** The Connectome Harmonic signature of DMT (energy difference) is shown alongside corresponding signatures of psilocybin and LSD-induced states previously reported in ref. [5], to enable visual comparison. The control placebo condition from the DMT study is also shown, to demonstrate that effects are specific to altered states of consciousness. **B** Fixed effects (and 95% CI) of projections (dot product) between the multivariate connectome harmonic signature of DMT, and four other states previously investigated by Luppi et al. [13] : anaesthesia (blue), DOC patients (violet), ketamine (orange), and LSD (green); all *p* < 0.00001.

DMT is a classical serotonergic psychedelic, pharmacologically related to psilocybin and LSD. Here, we show that the connectome harmonic signature of DMT coincides with the previously reported signatures of LSD and psilocybin [5, 12]. In Fig. 3A, we report the energy difference spectrum of CH overlaid with previously reported findings on LSD [5] and psilocybin [12]. The visual comparison allows to appreciate the similar pattern the decrease in low-frequency connectome harmonics and increase of high frequency harmonics for all the three classical serotonergic psychedelics (DMT, LSD and Psilocybin).

Importantly, the original analyses on the energy difference spectrum of CH considered each CH bin in isolation. However, it is clear that the overall pattern that emerges from considering all bins together is just as meaningful—if not more so. To take into account the full spectrum of connectome harmonic changes at the same time, we followed our previous work [13] and implemented Partial Least Squares Discriminant Analysis (PLS-DA): this data-driven technique allowed us to extract the multivariate patterns of connectome harmonic energy that maximally distinguish between DMT and placebo (termed “MultiVariate Signatures”, MVS). Here, we tested whether DMT would align positively with the multivariate signatures of LSD and psychedelic doses of ketamine, and negatively with the signatures of unconsciousness (awake vs propofol, and DOC fMRI+ vs fMRI-, corresponding to brain-injured patients who can (DOC fMRI+) versus cannot (DOC fMRI-) provide in-scanner evidence of responding to linguistic commands). We projected each subject’s connectome harmonic energy spectrum onto a given MVS (thereby measuring the correspondence between them) and then compared the value of this projection across DMT and placebo conditions. We clearly found that the multivariate connectome harmonic signature that best distinguishes DMT from placebo (DMT vs placebo), coincides with the analogous signatures of LSD (LSD vs placebo *p* < 0.00001) and psychedelic ketamine (ketamine vs placebo, *p* < 0.00001). Conversely, the DMT signature (DMT vs placebo) is the opposite of the signatures obtained by comparing wakefulness against propofol anaesthesia (awake vs propofol, *p* < 0.00001), or fMRI-responsive versus unresponsive DOC patients (DOC fMRI+ vs fMRI−, *p* < 0.00001) (Fig. 3B). Furthermore, these results are reproduced when using the 985 HCP participants connectome as the structural basis (Supplementary Fig. 7).

### DMT enhances the diversity of connectome harmonics repertoire

The prominent entropic brain account of psychedelic action posits that psychedelics exert their subjective effects at least in part by increasing the diversity (entropy) of spontaneous brain activity and connectivity, which would then translate to greater richness of subjective experience [19, 25] or ’phenomenal consciousness’ [26].

**Fig. 4:**
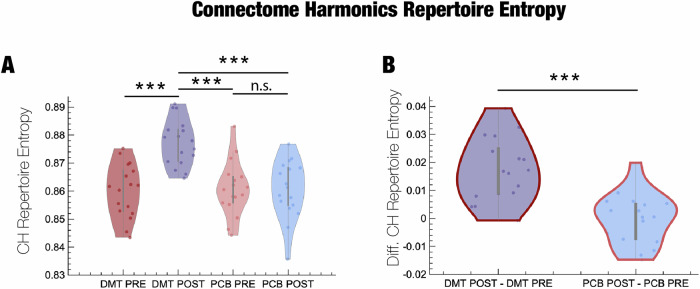
Repertoire of connectome harmonics and CH repertoire entropy. Repertoire entropy of connectome harmonics can be low if dominated by a specific range of spatial frequencies, and conversely the highest if the distribution of the spatial frequencies approaches the uniform distribution with maximum entropy. **A** CH repertoire Entropy (Pre/Post DMT: *p*-value = 0.0001, Pre PCB/Post DMT: *p*-value = 0.0003, Post PCB/Post DMT: *p*-value < 0.0001 and non-significant difference between Pre/Post PCB: *p*-value = 0.9278, paired *t*-test). **B** CH repertoire Entropy Difference (Diff. in Pre-Post DMT and Pre-Post PCB: *p*-value < 0.00001, paired t-test, ***p-value ≤ 0.0001).

Here, we therefore investigate whether DMT, a psychedelic, also increases the entropy of the connectome harmonic repertoire, as predicted by the entropic brain hypothesis and shown here in EEG data, where the entropy of spontaneous brain activity and subjective ’richness’ were strongly correlated [18]. We computed the normalised CH repertoire entropy for each condition (pre/post DMT and pre/post placebo) (Fig. 4). CH repertoire entropy increased for the other three conditions (Pre/Post DMT: *p*-value = 0.0001, Pre/Post PCB: *p*-value = 0.9278, Pre PCB/Post DMT: *p*-value = 0.0003, Post PCB/Post DMT: *p*-value < 0.0001, paired *t*-test) (Fig. 4A). Furthermore, the result was strengthened by comparing the CH repertoire entropy difference between post/pre DMT and post/pre placebo where an increase was observed as well (Diff. in Pre-Post DMT and Pre-Post PCB: *p*-value < 0.00001, paired *t*-test) (Fig. 4B). Furthermore, the changes in CH repertoire entropy of CH under DMT are robust to the choice of connectome as the results are consistent with the analysis performed with the 985 HCP participant connectome (Supplementary Fig. 3).

### Time-resolved coupling of harmonic signatures and subjective experience

Here, we wanted to address whether the changes in connectome harmonic signatures (CH repertoire entropy and energy spectrum difference) are related to changes in subjective experience in a time-resolved manner.

**Fig. 5:**
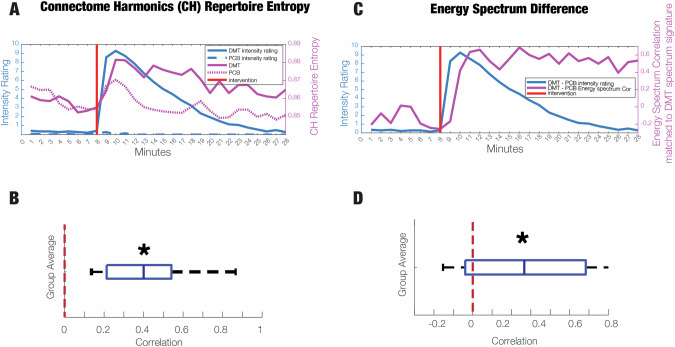
Time-resolved and subject-specific measures of CH repertoire entropy and energy spectrum difference in DMT. **A** The timecourse of CH Repertoire Entropy for the 28 min of recording. **B** Subject specific correlations between the CH Repertoire timecourses of the DMT condition and intensity ratings. We report the group average of the correlation values between CH Repertoire Entropy and Intensity Ratings is statistically significant from zero (black star, *p* = 0.00002). **C** The timecourse of Energy Spectrum Difference for the 28 min of recording. **D** Subject specific correlations between the Energy Spectrum Difference timecourses and intensity ratings. We report the group average of the correlation values between CH Repertoire Entropy and Intensity Ratings is statistically significant from zero (black star, *p* = 0.013).

First, we analyse whether temporal changes in the entropy of connectome harmonics correlate with temporal changes in the subjective rating of intensity of the DMT experience. We find that this is indeed the case: for half of the individuals, we found significant correlations between the intensity ratings and repertoire entropy of CH of the DMT session. We quantified it at a group level where these individual correlations are statistically significant from zero (t-test *p *= 0.00002, Fig. 5A, B). In other words, the changes in repertoire entropy of CH induced by DMT at the neural level, correlate with DMT-induced changes in subjective intensity.

Second, we investigate whether the ability to detect the energy spectrum difference signature of the psychedelic experience (shared by DMT with LSD and psilocybin) correlates with a more intense subjective experience. Once again, we find that this is the case: for five individuals, the correlations between energy spectrum difference and intensity ratings were significant. Importantly, we quantified these correlations at the group level where they were significant from zero (t-test *p* =  0.013, Fig. 5C, D). In line with CH repertoire entropy, the energy spectrum difference reflects the DMT-induced changes in subjective intensity in a time-resolved manner. We include the time-resolved evolution of the energy spectrum difference in the supplementary information (Supplementary Fig. 5).

Traditionally, EEG signatures as described by Lempel-Ziv (LZ) complexity [27, 28] have been shown to reflect well the DMT-induced subjective intensity in a time-resolved manner [18]. Here, we wanted to see whether we observe a cross-modal relationship between the different measures of complexity: namely the LZ complexity derived from EEG and CH repertoire entropy from fMRI. We show that indeed it is the case that on the group level the LZ complexity (defined as the difference between DMT and PCB conditions as well as DMT alone) correlate significantly with the CH repertoire entropy which is not the case for the placebo condition (Spearmann correlation **p < 0.001, ***p < 0.0001, Supplementary Fig. 4). However, when comparing ”richness of the experience” on a subject level to the magnitude of CH repertoire entropy, we have not observed significant correlation as has been previously shown between ”richness of the experience” and LZ complexity [18] (Supplementary Fig. 9).

### Sensitivity and robustness

To ensure the robustness of our results, we replicate our main analysis of DMT CH signature and match with other signatures using 25 logarithmically spaced bins instead of the 15 bins canonically employed for CHD analysis (Supplementary Fig. 6).

Conversely, we also show that this ability to replicate results is not merely an indicator that *any* basis function will produce similar results. We illustrate this point by using connectome harmonics obtained from a degree-preserving randomisation of the original structural connectome, which fails to show the loss of energy at low frequencies (Supplementary Fig. 8A), and fails to capture the expected relationship of DMT with ketamine and disorders of consciousness (Supplementary Fig. 8B).

## Discussion

We used connectome harmonic decomposition to represent functional brain signals in terms of their relationship with the detailed network organisation of the human connectome. We sought to understand how this structure-function relationship is altered by the potent psychedelic agent, DMT. Here, for the first time, fMRI recordings of participants under the influence of the psychedelic DMT were analysed with this method. The results demonstrate full harmonic spectrum changes under the influence of DMT, with a suppression of low-frequency harmonics and an increase of high frequency harmonics – consistent with previous findings with different psychedelics (psilocybin, LSD and ketamine). Furthermore, our results revealed an increase in CH repertoire entropy which is also in line with previously reported findings on other psychedelics (psilocybin, LSD and ketamine). Interestingly, both of these markers (Energy Spectrum Difference and CH repertoire entropy) tracked the DMT experience in a time-resolved manner and coupled to the subjective experience of individual participants.

The entropic brain hypothesis proposed that the richness of the spatio-temporal dynamics can be quantified in terms of entropy, which is considered to index the richness of conscious experience. Furthermore, it proposed and later showed that the psychedelic-induced state would feature increase in the level of entropy within the brain [29–31] (but see the following work for a comprehensive assessment of different entropy measures under psychedelics [32]). Here, we have shown, for the first time, the effect of DMT on repertoire entropy as defined by the connectome harmonic power spectrum. The increase that we observed – which is consistent with the entropic brain hypothesis and with previous psychedelic findings [13] is supported by an increase in the high- frequency energy spectrum of harmonic contributions, and, at the same time, a suppression of the low frequency energy spectrum - representative of global contributions from the large-scale structural connectivity [5, 6, 12].

How structure shapes function has been at the forefront of contemporary neuroscience [2, 33] with many approaches considered [34–37]. Recent advances have considered diffusion process to describe the unfolding brain activity on the structural connectome, of which connectome harmonics are the representative example [6], but also considered elsewhere [38–41]. Also, approaches based on different communication models have been explored [21, 42]. In general terms, the correlation strength of structure-function relationships has been indicative of the level of consciousness - a stronger relationship has signified a loss of consciousnesses, for example in anesthesia [8, 9, 43]. In terms of connectome harmonics one of the potential interpretations has been that low frequency harmonics approximate the global structural topology of the underlying graph, while higher-frequency harmonics capture localised representations. This is relevant, as the observed effect here is the opposite to the reduced levels of consciousness, with a suppression of lower frequency harmonics and an increase of high frequency harmonics suggesting an opposite trend in reduced levels of consciousness towards a brain state governed by the global (rather than local) organisation of the structural connectome. This has been explored in a recent study where high generalisibility of the connectome harmonic decomposition spectrum was shown across minimal conscious, anesthetic, and ketamine and LSD-induced psychedelic states [13]. Meaning, CHD spectrum could be used to categorize these diverse states of consciousness in a predictable and meaningful way. Indeed, the present study also found that the DMT harmonic signature is analogous to the ones elicited by LSD and ketamine, and opposite to the signatures of anaesthesia and disorders of consciousness.

To represent fMRI activity in different brain states, it is possible to use different bases on which the activity is projected. Indeed, recent work and an ongoing debate have highlighted the importance of geometry as a key structural feature in shaping the unfolding dynamics [23, 44–46]. In this sense, connectome harmonics can be viewed as an extension of spherical harmonics - similarly derived as the eigenfunctions of the Laplace operator applied to the sphere [23, 47, 48]. Hence, when considering only local grey-matter connectivity, connectome harmonics reflect spherical harmonics represented on the cortical surface. However, we argue here that rare long-range connectivity is a necessary feature for an accurate representation of brain states [24, 49]. Therefore, connectome harmonics are extending spherical harmonics approaches by embedding both local grey matter and long-range white matter connectivity of the human brain. Moreover, when the underlying graph is randomised (even as the number of connections of each node is preserved), the ability to correctly identify brain states corresponding to the psychedelic state versus loss of consciousness is lost (Supplementary Fig. 8) consistently with what was previously observed [50].

Experimentally, the DMT dataset is a single-blind, counter-balanced and placebo-controlled design and contains a control group that is important to differentiate the changes in the connectome harmonic decomposition under the influence of DMT from its baseline. Moving forward, future work might further differentiate the level of vigilance that comes with the psychedelic experience by considering additional control groups under the influence of stimulants such as modafinil and caffeine. Recently, this was done with methylphenidate, controlling for arousal. Methylphenidate matched psilocybin in its pro-arousal effects but failed to show the marked characteristic brain function changes [51]. Lastly, the dataset size of 17 participants (and 14 for the time-resolved analysis) reflects both drop-out rate (5 participants) and motion-artefact removal (3 participants plus additional 3 participants for the time-resolved analysis) which is a limitation for the power of the study and its subsequent statistics. Nonetheless, this limitation is partly compensated by the strong and reproducible effects elicited by DMT on both the brain and subjective experience.

## Supplementary information

N,N-dimethyltryptamine effects on connectome harmonics, subjective experience and comparative psychedelic experiences

## Data Availability

Source data for the Figs. 2–5 are shared in the OSF open access data repository - https://osf.io/4ep8g/. Raw data analysed during the current study are available on request from the following authors. Psilocybin, LSD and DMT datasets: Dr. Robin L. Carhart-Harris (Imperial College London/University of California – San Francisco; email: robin.carhart-harris@ucsf.edu).
