# Supplementary material for: N,N-dimethyltryptamine effects on connectome harmonics, subjective experience and comparative psychedelic experiences
Source: Neuropsychopharmacology. 2025 Sep 12;50(12):1768–76. doi: 10.1038/s41386-025-02190-4 (PMC12518839; doi:10.1038/s41386-025-02190-4)
Supplement: Supplementary file 1 — N,N-dimethyltryptamine effects on connectome harmonics, subjective experience and comparative psychedelic experiences [file 41386_2025_2190_MOESM1_ESM.pdf]

Supplementary Material: Time-resolved coupling between connectome harmonics and subjective experience under the psychedelic DMT

Jakub Vohryzek[1,2,11], Andrea I. Luppi[1,3,4,5,11], Selen Atasoy[1,6], Gustavo Deco[2,7], Robin L. Carhart-Harris[8,9], Christopher Timmermann[8,10], Morten L. Kringelbach[1,3,6]

[1]Centre for Eudaimonia and Human Flourishing, Linacre College, University of Oxford, Oxford, United Kingdom
[2]Center for Brain and Cognition, Computational Neuroscience Group, Department of Information and Communication Technologies, Universitat Pompeu Fabra, Barcelona, Spain
[3]Department of Psychiatry, University of Oxford, Oxford, United Kingdom
[4]St John's College, University of Cambridge, Cambridge, United Kingdom
[5]Division of Information Engineering, University of Cambridge, Cambridge, United Kingdom
[6]Center for Music in the Brain, Aarhus University, Aarhus, Denmark
[7]Centre for Psychedelic Research, Department of Brain Sciences, Imperial College London, London, United Kingdom
[8] Institució Catalana de la Recerca i Estudis Avançats (ICREA), Passeig Lluís Companys 23, Barcelona, 08010, Spain
[9]Departments of Neurology and Psychiatry, University of California San Francisco, San Francisco, USA
[10]Department of Experimental Psychology, University College London, London, UK
[11]These authors contributed equally: Jakub Vohryzek, Andrea Luppi

Materials and Methods

Psilocybin Dataset

A complete description of the psilocybin study protocol can be found in the original paper. In brief, nine participants were considered for this analysis following rigorous exclusion criteria; no younger than 21 year of age, pregnancy, history of psychiatric disorders, cardiovascular disease, substance dependence, claustrophobia, blood or needle phobia or adverse response to psychedelics. Furthermore, participants were excluded if their mean framewise displacement (FD) exceeded 0.4 mm. Two eye-closed scans, separated by seven days, were performed for each participant. In a counterbalanced design each participant was given intravenously either psilocybin (2 mg dissolved in 10 ml saline, 60 s) or saline (10 ml saline, 60 s). The recording session lasted 12 minutes with the 6th minute marking the solution infusion. For the study last five minutes (post the 60 s infusion) were analysed and a control pre-injection recording was matched accordingly to be 5 minutes as done in the original study. We analysed segments of 100 timepoints pre- and post-psilocybin injection (TR = 3 seconds).

LSD Dataset

A complete description of the LSD study protocol can be found in the original paper. Briefly, fifteen subjects were considered for this analysis with key exclusion criteria; no younger than 21 year of age, pregnancy, history of psychiatric disorders, cardiovascular disease, substance dependence, claustrophobia, blood or needle phobia or adverse response to psychedelics, previous experience with serotonergic psychedelics or use within 6 weeks of first scanning. Furthermore participants were excluded if their mean framewise displacement (FD) exceeded 0.4 mm. In a counter-balanced design each participant was given intravenously either LSD (75 μ g in 10 ml saline, 120 s) or saline (10 ml saline, 120 s). After 60 minutes acclimatisation in the scanner, there were three resting-state recording sessions; one with music interleaved in-between two standard resting-state sessions. Only results of the first resting-state recording session are considered here as done in the original study. The scan lasted 7.23 minutes with a TR of 2 seconds, resulting in 217 timepoints pre- and post-LSD injection.

High-resolution alternative reconstruction of the human connectome

To demonstrate that our results are not fundamentally dependent on this specific operationalisation of the human connectome, we also used an alternative representative human connectome. The alternative connectome was constructed from multi-shell diffusion-weighted imaging data from 985 subjects of the HCP 1200 data release (`http://www.humanconnectome.org/`), each scanned for approximately 59 minutes. This represents a nearly 100-fold increase in sample size compared with the original connectome used for connectome harmonic decomposition by Atasoy and colleagues. We refer to the human connectome constructed from these data as the HCP-985 connectome. Acquisition parameters are described in detail in the relative documentation (`http://www.humanconnectome.org/`), and the dMRI data were preprocessed and made available as part of the freely available Lead-DBS software package (`http://www.lead-dbs.org/`). For the reconstruction of long-range white matter tracts of each individual, we used the following procedure: the diffusion data were processed using a generalized q-sampling imaging algorithm implemented in DSI Studio (`http://dsi-studio.labsolver.org`). A

white-matter mask was obtained from segmentation of the T2-weighted anatomical images, which were co-registered to the b0 image of the diffusion data using SPM12. In each HCP participant, 200,000 fibres were sampled within the white-matter mask, using a tracking method that previously achieved the highest valid connection score among 96 methods submitted from 20 different research groups in a recent open competition. Finally, the fibres were transformed into standard Montreal Neurological Institute (MNI-152I) space using Lead-DBS software. The remaining procedures for obtaining individual connectomes and aggregating them into a group-average representative connectome, and subsequent connectome harmonic decomposition, were the same as described above.

Randomised connectome

To demonstrate the importance of the specific topology of the human connectome, obtained by combining local grey matter connectivity and long-range white matter fibres, we also tested whether our results would replicate when using harmonics obtained from a randomised connectome. Before performing Laplacian decomposition (as described in the next section), the original connectome was therefore turned into a random network using the degree-preserving procedure implemented in the Brain Connectivity Toolbox. Harmonics were then extracted from Laplacian eigendecomposition, and the full connectome harmonic decomposition pipeline was followed.

Derivation of Connectome Harmonics

Connectome Harmonics were computed from the structural connectome described as a graph, $\Re = (\nu, \varepsilon)$, of vertices $\nu = \{v_i | \in 1, ..., n\}$ and edges $\varepsilon = \{e_{ij} | \in \nu \times \nu\}$. The graph's edges represent 1) local connectivity, defined by 6 nearest neighbours of each vertex on the cortical surface, and 2) long-range connectivity as determined by tractography in terms of cortico-cortical fibres. The graph's edges were further binarised and symmetrised and represented in an unweighted and undirected adjacency matrix, A, as follows:

$$A(i,j) = \begin{cases} 1, & \text{if } (i,j) \in \varepsilon \\ 0, & \text{otherwise} \end{cases} \tag{1}$$

where i,j are the indices of the adjacency matrix A (20,424 $\times$ 20,424). In order to obtain a group adjacency matrix $\bar{A}$, the individual adjacency matrices of the 10 subjects were averaged. Furthermore, the discrete counterpart of the Laplace operator Δ, applied to the structural connectivity (local and long-range) of the human brain $\bar{A}$, is estimated by computing the symmetric Laplacian $\Delta_{\bar{A}}$ in the following manner:

$$\Delta_{\bar{A}} = D^{-1/2}\, L\, D^{-1/2}, \text{ where } L = D - \bar{A} \tag{2}$$

where the D is the diagonal degree matrix, $D = \sum_{i=1}^{n} \bar{A}(i,j)$. Lastly, the connectome harmonics,ψ_k, $k \in 1, ..., n$ were defined and computed as the eigenvectors of the following eigenvalue problem,

$$\Delta_{\bar{A}} \psi_{k(v_i)} = \lambda \psi_{k(v_i)}, \forall\, v_i \in \nu \tag{3}$$

where $\lambda, k \in 1, ..., n$ are the associated eigenvalues of $\Delta_{\bar{A}}$.

Connectome Harmonic Decomposition of Resting-state fMRI

The derived connectome harmonics can be used to represent resting-state fMRI in each of the DMT conditions. First, resting-state fMRI is projected from its MNI voxel-space to the Freesurfer surface vertex-space using the HCP command *-volume-to-surface-mapping*. The resulting timecourse can be represented as $\mathcal{F}(v,t)$ for every vertex $v \in \nu$. Once in the same space, the fMRI activity $\mathcal{F}(v,t)$ at every time step $t \in [1,...,T]$ can be described as a sum of weighted contributions α_k of individual connectome harmonics k. This fMRI decomposition can be described in the following format,

$$\mathcal{F}(t_i) = \sum_{k=1}^{n} \alpha_k(t_i)\psi_k = \alpha_1(t_i)\psi_1 + \alpha_2(t_i)\psi_2 + ... + \alpha_n(t_i)\psi_n \tag{4}$$

with α_k being the contribution of k connectome harmonic ψ_k to the fMRI activity $\mathcal{F}(t_i)$ at time t_i. Formally, the connectome harmonic contributions are described as $\alpha_k(t) = \langle \mathcal{F}(t), \psi_k \rangle$.

Measures

Further analysis describes several measures which summarise different aspects of the decomposed fMRI timecourses in terms of the contributions of the connectome harmonic spectrum.

Power and Energy

The connectome harmonic weight α_k at each time t represents the strength of a given connectome harmonic ψ_k of that particular fMRI pattern $\mathcal{F}(v,t)$ at the same time t and its absolute value can be defined as power, $P(\psi_t, t) = |\alpha_k(t)|$. To further estimate the connectome harmonic contribution α_k in relation to its eigenvalue λ_k, a measure of energy, E, is defined as the square of connectome harmonic contribution and its intrinsic energy,(λ), in the following way $E(\psi_k, t) = |\alpha_k(t)|^2\lambda_k^2$. Note that both Power and energy are computed for every connectome ψ_k at every timepoint t across the fMRI timecourse $\mathcal{F}(v,t)$. Moreover,we used the energy spectrum averaged over 15 bins of varying logarithmic scale for the 18k connectome harmonics. Subsequently, the energy spectrum is defined as the difference between the 8 minutes recordings of the pre- and post-recording sessions for the DMT and placebo groups.

To ensure that our results are not specific to the chosen number of bins, we also replicate our main analysis using 25 bins instead of 15. Additionally, to ensure that our results were not unduly influenced by potential aliasing effects introduced by the use of high-resolution diffusion data, for the HCP-985 analyses we only used the first 14 logarithmically spaced bins (instead of 15 as for the previous analyses), showing that our results are not critically dependent on the precise number of bins. Likewise, for the analysis with randomised connectome we also only used the first 14 logarithmically spaced bins.

Repertoire Entropy of Connectome Harmonics

The repertoire entropy of CH, H, was defined as the time-averaged histogram entropy of the binned (15 logarithmic bins) power distribution, P, at each condition i.e.

$$H(t_i) = -\sum_{1}^{k} p(P(\psi_k, t_i))log_2 p(P(\psi_k, t_i)). \tag{5}$$

The bin choice for both measures was chosen historically as a common frame of reference for comparison with previous literature. Intuitively, the flatter the distribution the higher the repertoire entropy of connectome harmonics, while the more concentrated distribution to a given frequency range the lower the repertoire entropy of connectome harmonics.

Data-driven extraction of multivariate connectome harmonic signatures

Partial least squares (PLS) is a multivariate statistical analysis used to identify relationships between one or more targets (Y) and a set of predictor variables X. This method extracts principal components as linear combinations of variables in each set that maximally covary with each other. In the present case, for both DMT and placebo, X was the matrix of 15 binned energy values (see above) for each individual (averaged over timepoints), and Y was the vector of binary classification between the two states (DMT or placebo) – making this an application of Partial Least Squares Discriminant Analysis (PLS-DA), owing to the binary nature of Y. The first principal component extracted by PLS-DA represents the single most discriminative pattern present in the data, in terms of distinguishing observations (subjects) belonging to the two different classes (placebo versus DMT).

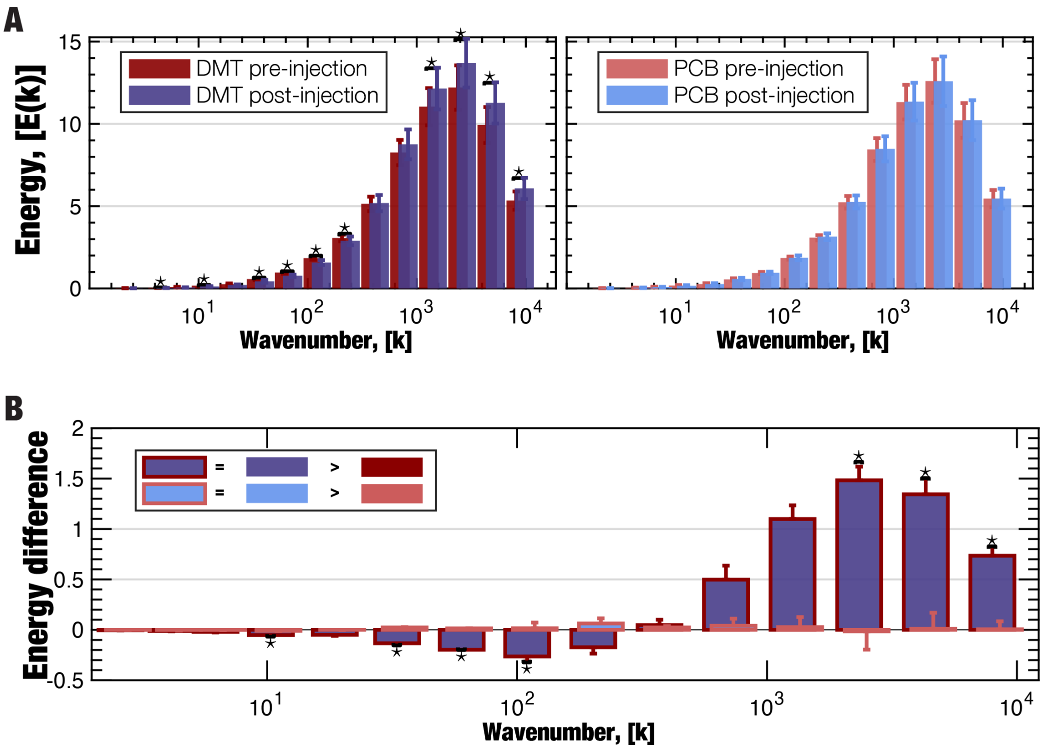

Fig S1 **A** Replication of DMT CH energy signature using dense structural connectome from 985 HCP participant. **B** Replication of DMT CH energy difference signature using dense structural connectome from 985 HCP participant.

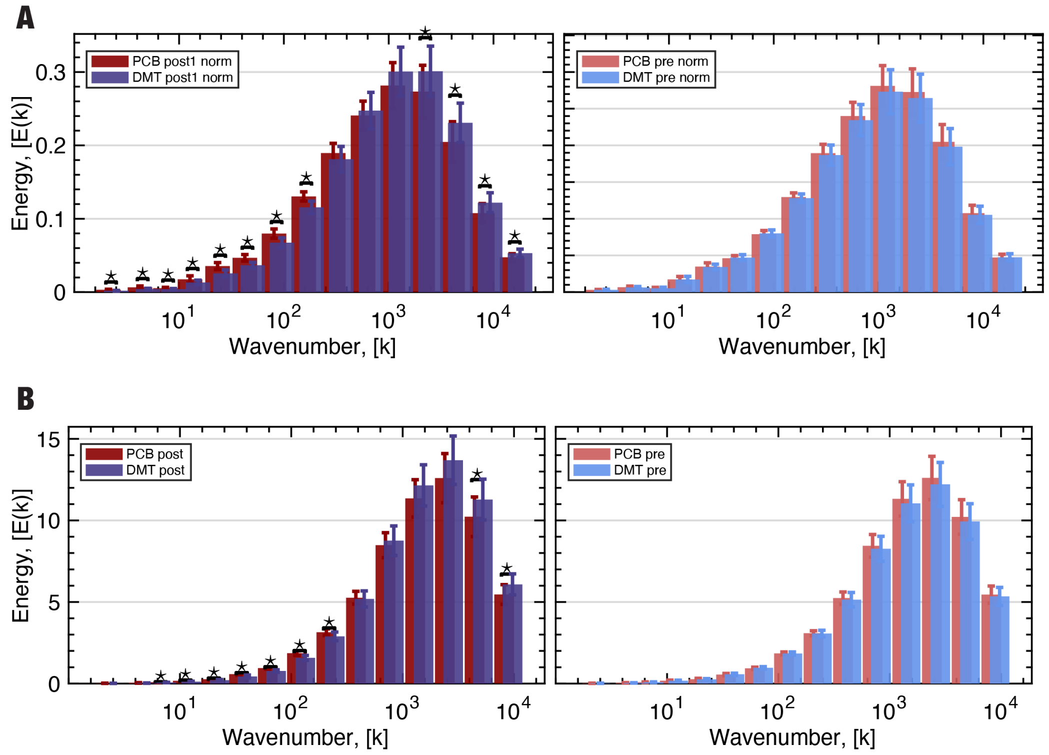

Fig S2 **A** The comparison of post DMT conditions of CH energy signature using the original connectome. **B** Replication of the post DMT condition of CH energy signature using dense structural connectome from 985 HCP participant.

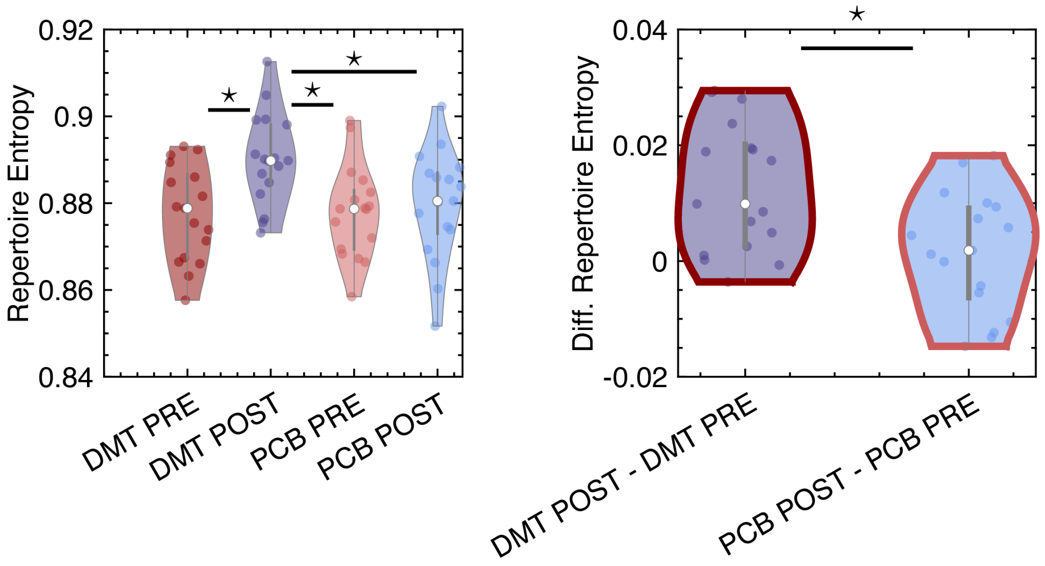

Fig S3 A Replication of DMT CH repertoire entropy using dense structural connectome from 985 HCP participant. Repertoire Entropy (Pre/Post DMT: p-value $< 10^{-4}$, Pre PCB/Post DMT: p-value $< 10^{-4}$, Post PCB/Post DMT: p-value $< 10^{-4}$ and non-significant difference between Pre/Post PCB, paired t-test). Repertoire Entropy Difference (Diff. in Pre-Post DMT and Pre-Post PCB: p-value $< 10^{-5}$, paired t-test).

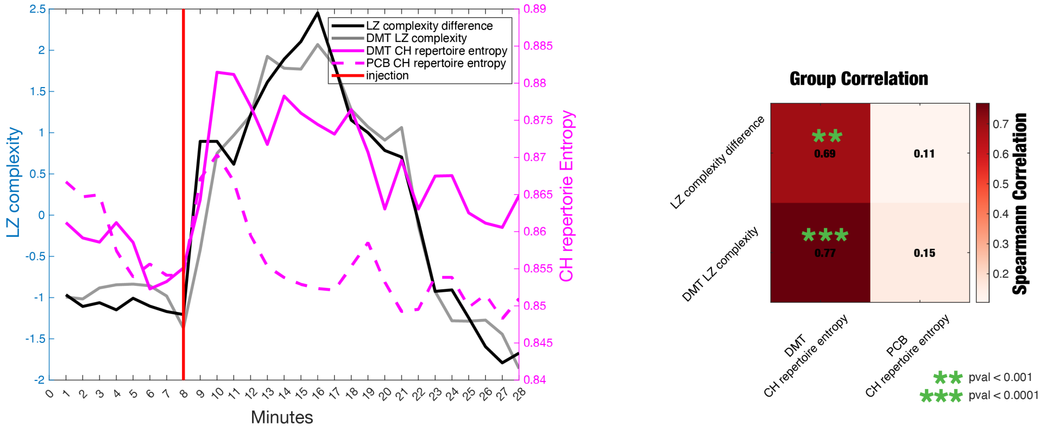

Fig S4 FMRI-based CH repertoire entropy correlates with EEG-based LZ complexity. Both the subject-averaged DMT LZ complexity and DMT-PCB complexity biomarkers from Timermann et al. (2023) significantly correlate with the CH repertoire entropy measures of this study (LZ complexity difference vs. DMT CH repertoire entropy p-val <0.001, DMT LZ complexity vs. DMT CH repertoire entropy p-val <0.0001)

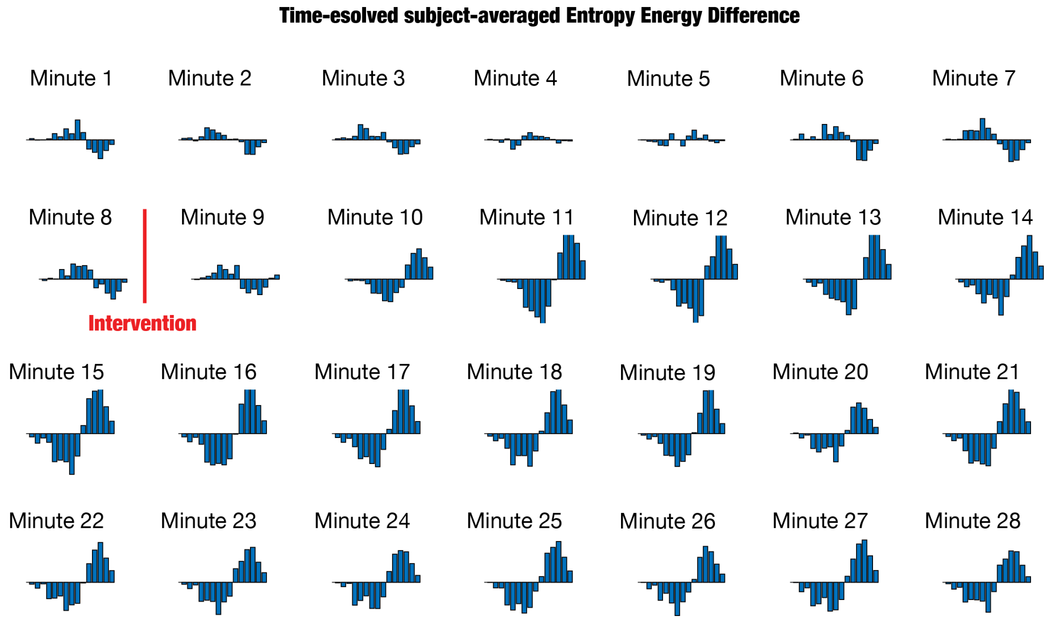

Fig S5 Time-unfolded Energy Spectrum Difference. Time-resolved measure of Energy Spectrum Difference across the 28 minutes of recording averaged across all the subjects

Bin	DMT	PCB	DMT vs. PCB
1	0.000111534153677	0.443739045406381	0.000072039040152
2	0.000096264112382	0.978653551327367	0.000590445704116
3	0.001233725098961	0.754643048397676	0.002604900707335
4	0.003077022951818	0.877283528058312	0.009994441646813
5	0.000000256236014	0.509275956413800	0.000002058362231
6	0.000000396679874	0.560649073886066	0.000000684459921
7	0.000000102977299	0.469780426882757	0.000016228702831
8	0.000000986752337	0.545800502470496	0.000266441593202
9	0.116931684833190	0.909095948550599	0.191318048943926
10	0.017108101927998	0.773940449012562	0.058560020173660
11	0.001145208353094	0.930154024173996	0.006960773952193
12	0.000138555925145	0.909870228487999	0.002333338374682
13	0.000076037644482	0.961990017577697	0.000691256275394
14	0.000044367052205	0.978568040573966	0.000745873942288
15	0.000077737866835	0.772433833032214	0.001271892584016

Table S1 Paired t-test p-values for Figure 2. We report all the comparisons for DMT pre-injection versus DMT post-injection, PCB pre-injection versus DMT post-injection and ΔDMT vs. ΔPCB.

A

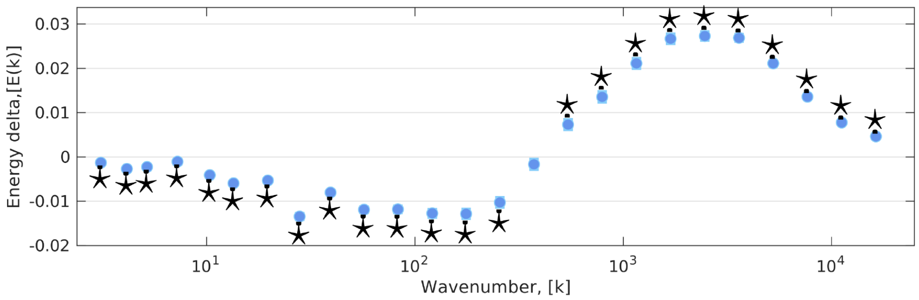

B

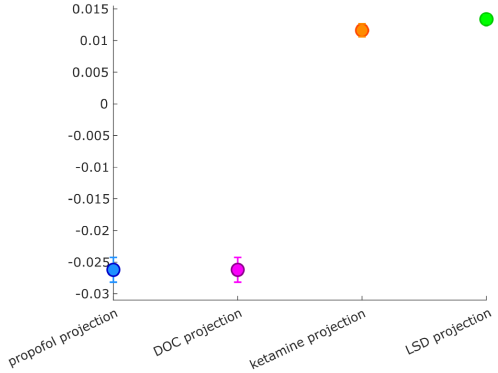

Fig S6 **A** Replication of DMT CH energy signature using 25 bins. **B** Replication of the relationship between multivariate CH signatures of DMT and other states of altered consciousness, using 25 bins. Plots show the fixed effects (and 95% CI) of projections (dot product) between the multivariate connectome harmonic signature of DMT, and four other states previously investigated by Timermmann et al. (2023): anaesthesia (blue), DOC patients (violet), ketamine (orange), and LSD (green).

A

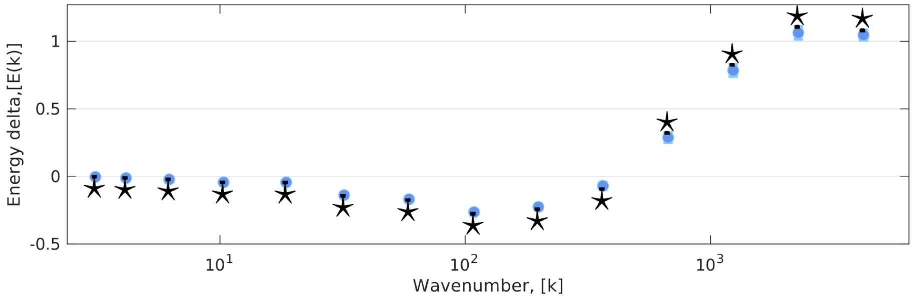

B

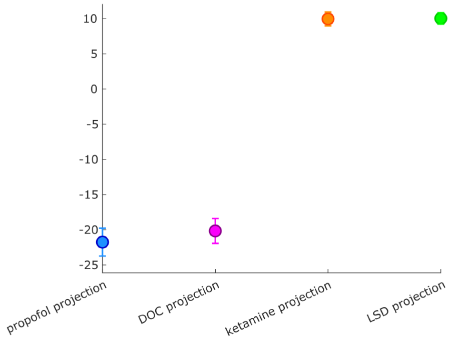

Fig S7 **A** Replication of DMT CH energy signature using a high-resolution human connectome from N=985 healthy individuals. **B** Replication of the relationship between multivariate CH signatures of DMT and other states of altered consciousness, using a high-resolution human connectome from N=985 healthy individuals. Plots show the fixed effects (and 95% CI) of projections (dot product) between the multivariate connectome harmonic signature of DMT, and four other states previously investigated by Luppi et al. (2023): anaesthesia (blue), DOC patients (violet), ketamine (orange), and LSD (green).

A

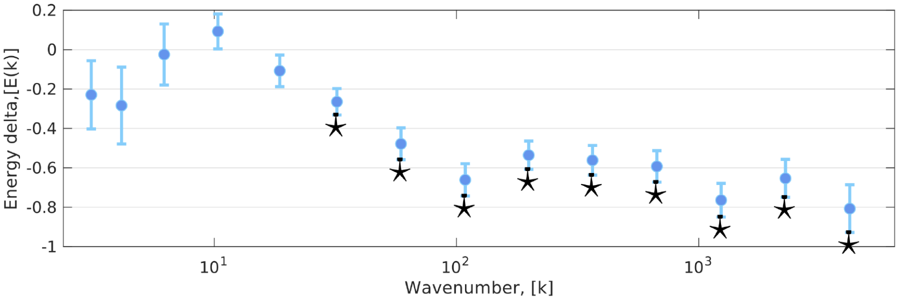

B

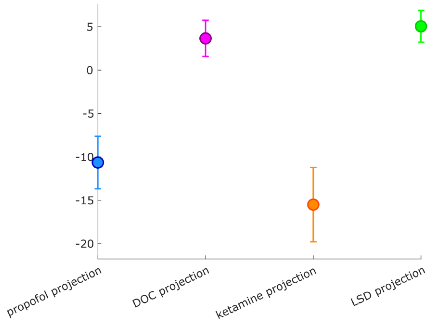

Fig S8 A Replication of DMT CH energy signature using connectome harmonics from a degree-preserving randomised connectome. **B** Replication of the relationship between multivariate CH signatures of DMT and other states of altered consciousness, using connectome harmonics from a degree-preserving randomised connectome. Plots show the fixed effects (and 95% CI) of projections (dot product) between the multivariate connectome harmonic signature of DMT, and four other states previously investigated by Luppi et al. (2023): anaesthesia (blue), DOC patients (violet), ketamine (orange), and LSD (green).

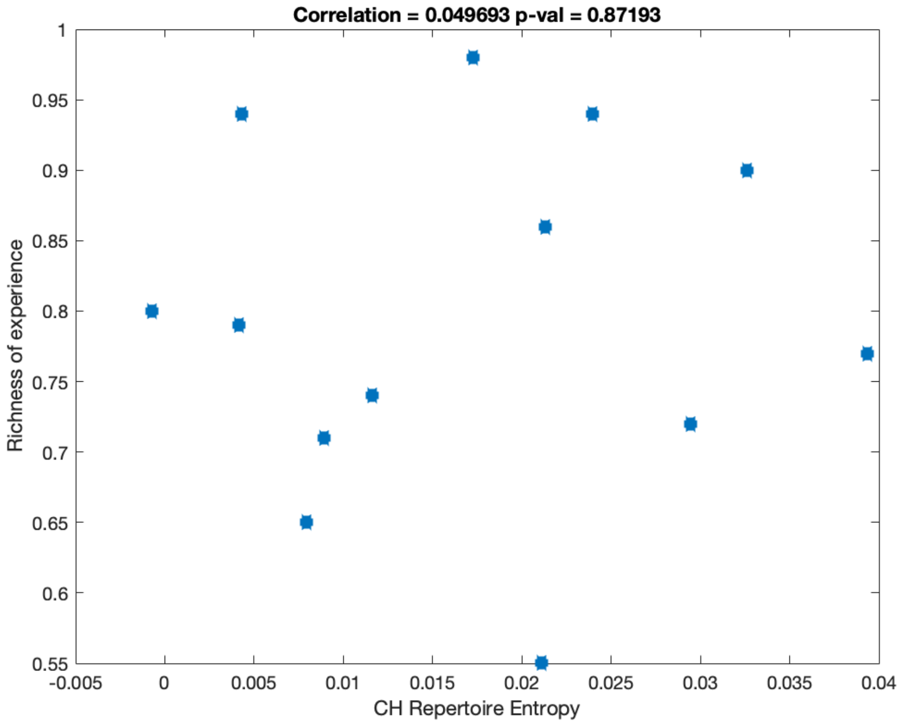

Fig S9 A Correlation between CH Repertoire Entropy and ”richness of the experience” under the DMT-induced state. Unlike in Timmernamnn et al. (2023) where LZ complexity has been associated with ”richness of the experience”, here we report a non-significant Spearmann correlation between CH Repertoire Entropy and ”richness of the experience” (corr = 0.05, p-val = 0.87).
